# Innate Immune Function in Placenta and Cord Blood of Hepatitis C – Seropositive Mother-Infant Dyads

**DOI:** 10.1371/journal.pone.0012232

**Published:** 2010-08-30

**Authors:** Christine Waasdorp Hurtado, Lucy Golden-Mason, Megan Brocato, Mona Krull, Michael R. Narkewicz, Hugo R. Rosen

**Affiliations:** 1 Section of Pediatric Gastroenterology, Hepatology and Nutrition, Department of Pediatrics Digestive Health Institute, University of Colorado School of Medicine, The Children's Hospital, Aurora, Colorado, United States of America; 2 Division of Gastroenterology and Hepatology, University of Colorado School of Medicine, Aurora, Colorado, United States of America; 3 Integrated Program in Immunology, University of Colorado and National Jewish Hospital, Denver, Colorado, United States of America; 4 Department of Obstetrics and Gynecology, Denver Health Medical Center, Denver, Colorado, United States of America; Karolinska Institutet, Sweden

## Abstract

Vertical transmission accounts for the majority of pediatric cases of hepatitis C viral (HCV) infection. In contrast to the adult population who develop persistent viremia in ∼80% of cases following exposure, the rate of mother-to-child transmission (2–6%) is strikingly low. Protection from vertical transmission likely requires the coordination of multiple components of the immune system. Placenta and decidua provide a direct connection between mother and infant. We hypothesized that innate immune responses would differ across the three compartments (decidua, placenta and cord blood) and that hepatitis C exposure would modify innate immunity in these tissues. The study was comprised of HCV-infected and healthy control mother and infant pairs from whom cord blood, placenta and decidua were collected with isolation of mononuclear cells. Multiparameter flow cytometry was performed to assess the phenotype, intracellular cytokine production and cytotoxicity of the cells. In keeping with a model where the maternal-fetal interface provides antiviral protection, we found a gradient in proportional frequencies of NKT and γδ-T cells being higher in placenta than cord blood. Cytotoxicity of NK and NKT cells was enhanced in placenta and placental NKT cytotoxicity was further increased by HCV infection. HCV exposure had multiple effects on innate cells including a decrease in activation markers (CD69, TRAIL and NKp44) on NK cells and a decrease in plasmacytoid dendritic cells in both placenta and cord blood of exposed infants. In summary, the placenta represents an active innate immunological organ that provides antiviral protection against HCV transmission in the majority of cases; the increased incidence in preterm labor previously described in HCV-seropositive mothers may be related to enhanced cytotoxicity of NKT cells.

## Introduction

Hepatitis C virus (HCV) is the most common blood borne infection in the United States (US), with an overall prevalence of 1.8% [1] that varies according to racial and ethnic groups. The most striking clinical features of infection with HCV are its extraordinarily high propensity to develop persistent viremia (∼80%) [2], and the high proportion of patients who develop long-term complications including cirrhosis and liver failure [3], [4]. Approximately 1% of pregnant women have HCV infection [5], [6], corresponding to 40,000 births annually in the United States. With vertical transmission rates between 2–6% in women without HIV co-infection, vertical transmission accounts for the vast majority of pediatric HCV cases [7]. In light of the high rate of chronic infection developed in the non-pregnant state and the significant rate (15–35%) in HIV, the remarkably low rate of HCV infection following exposure in utero and at delivery, warrants further study [8], [9]. Protection from vertical transmission of HCV likely requires coordination of multiple components of the immune response including cell migration for surveillance and recognition of invading microorganisms [10]. Although the precise mechanisms as they pertain to HCV are still unclear, it is known that during normal pregnancy, the human decidua contains a high number of immune cells, including macrophages, T cells, and natural killer (NK) cells [10]. Clearly, the placental immune system represents an active immunological organ that functions critically as a regulator between the mother and the fetus and is capable of responding to pathogens [10]. Moreover, a placental infection that elicits the production of inflammatory cytokines such as IFN-γ and TNF-α could activate the maternal immune system, leading to placental damage and preterm labor [10]. In this regard, infection with HCV is a newly identified independent risk factor for preterm delivery, perinatal mortality, intrauterine growth restriction, and other complications [11]–[13].

The placenta provides the direct connection to maternal decidua and is separated from maternal blood supply by only one to three cells [14]. Despite this close connection, placental tissue from term infants has not been characterized with respect to immune composition or function. In contrast, decidua has been well characterized in association with fertility and fetal loss with the majority of the tissue examined from first and second trimester losses [15]–[17]. Notably, the decidual NK cell population consists of more CD56^bright^ than CD56^dim^, although the former population has been shown to decline during each trimester [18]. Functional evaluation has not shown decidua cytotoxicity to be elevated, despite an increase in activation markers and cytokine production [19], [20]. Cord blood from term infants has also been well-characterized in both immune composition and function due to the use of cord blood stem cells for bone marrow transplant [21], [22]. It is known that cord blood NK cells have decreased cytokine production compared to peripheral blood [22], [23].

Recognizing that pregnancy represents a unique immune condition and the apparent paradox of the low rate of HCV fetal transmission (compared to adults with acute infection), we hypothesized that the innate immune profiles would be different in placenta, decidua and cord blood and that HCV infection would modulate innate immunity at these sites. We thus undertook the first prospective study of the immune function of these components in pregnant women with chronic HCV infection and their infants.

## Results

### Study population

The study population was comprised of 12 treatment naïve HCV dyads (HCV-seropositive women and their infants) and 16 healthy pregnant non-HCV-seropositive control dyads. The populations did not differ significantly in maternal age, race/ethnicity, infant sex, infant gestational age or infant weight (p>0.17). The HCV dyad mothers had a median age of 30.5 years (20–45 years), 42% were Caucasian and 42% Hispanic. The infants in the HCV dyad group had a median gestational age of 39+3 weeks with a median birth weight of 3255 grams (range 1765–4560 grams). The control group was similar with mothers having a median age of 26 years (19–35 years), 31% were Caucasian and 56% Hispanic. Control infants had a median gestational age of 39+2 weeks with a median birth weight of 3440 grams (range 3100–4455 grams). The infants were equally divided between males and females in both the HCV and control groups. All HCV-infected mothers with identifiable virus (n = 8) were genotype 1a. Maternal viral loads measured the day of delivery are shown in **Table 1**.

**Table 1:** Maternal HCV (PCR) viral load.

Subject	HCV (IU/ml)	HCV Risk Factor
1	>7,692,310	Blood Transfusion
2	2,080,840	No Known
3	377,125	No Known
4	368,344	IVDU, Tattoo
5	328,662	IVDU, Tattoo
6	262,534	No Known
7	140,548	No Known
8	<43	Blood Transfusion
9	<43	No Known
10	<43	Blood Transfusion
11	<43	No Known
12	<43	IVDU

Viral load on the day of delivery with 5 mothers demonstrating HCV Ab positivity with HCV RNA negativity. Maternal HCV risk factors are listed with 50% having no known HCV risk factor or exposure.

IVDU  =  Intravenous Drug Use.

### Direct *ex vivo* lymphocyte composition of placenta

Fetal placental tissue has not previously been characterized with respect to overall lymphocyte composition, and information on the immune composition of term maternal decidual tissue is sparse. The innate immune system plays a central role in protection from viral infection and is therefore the primary focus of this analysis. In addition, this unique study cohort allowed us to study the impact of HCV exposure on these tissue lymphocytes.

The overall lymphocyte composition of placenta is remarkably similar to cord blood (CB) and decidua with only a few noted differences between tissues and HCV exposure groups (**Table 2**). Cord blood and maternal peripheral blood mononuclear cells (PBMC) also demonstrate similar compositions with differences only noted in T lymphocytes and natural killer T cells (NKTs) (**Table 2**). NK cells, central to innate immunity against viral pathogens, trend to higher levels in decidua than placenta and CB in both controls and HCV exposed subjects. NKT (CD3^+^CD56^+^) populations were found at greater frequencies within the placenta relative to CB in control and HCV-exposed infants (p<0.008). NKT cells increased in HCV-exposed placenta compared to control placenta (p = 0.04) (**Table 2**). B lymphocyte frequency trended higher in HCV exposed infants and HCV infected mothers. Specifically, B lymphocyte levels are higher in HCV exposed CB compared to controls (p = 0.04) with the highest levels detected in HCV decidua (**Table 2**).

**Table 2:** Lymphocyte composition of cord blood, PBMC, placenta and decidua in control and HCV dyads.

	T Lymphocytes (CD3^+^)	NK Cells (CD3^-^CD56^+^)	CD56+ NKT (CD3^+^CD56^+^)	B Lymphocytes (CD19^+^)	Macrophages (CD14^+^)	Total Dendritic Cells
Control Cord Blood	42.0%(20.9–79)	7.1%(2.3–24)	0.20%(0.1–0.7)	8.6%(4.3–24.0)	7.9%(3.1–21.2)	0.30%(0.42–1.38)
HCV Cord Blood	39.7(11.5–60)	6.9(2.7–12.4)	0.25(0.1–0.7)	12.5^4^(6.5–18.4)	7.2(3.1–11.4)	0.18(0.13–0.30)
HCV PBMC	69.5^1^(25.1–74.8)	10.3(2.4–41.4)	2.9(1.9–11.3)	14.5(7.2–17.6)	11.9(1.9–23.7)	NA
Control Placenta	43.7(30.9–58.8)	10.7^2^(5.4–20.3)	0.70^3^(0.1–1.3)	8.3(3.6–16.4)	5.7(2.8–44.8)	0.22(0.15–0.36)
HCV Placenta	45.3(21.7–50.2)	7.1(6.1–25.2)	1.1(0.6–1.5)	13.5(3.7–22.9)	5.7(2.1–9.2)	0.12(0.6–0.27)
Control Decidua	45.1(18.3–57.9)	19(13.5–33.2)	2.2(1.0–3.0)	12.2(10.1–33.6)	6.3(1.7–12.7)	NA
HCV Decidua	38(29.8–50.0)	18.9(8.3–21.9)	1.4(0.8–3.0)	19.3(13.5–25.7)	6.3(1.7–22.9)	NA

1 HCV PBMC T Lymphocytes higher than HCV cord blood (p = 0.02).

2 Decidua NKs higher percentage than Placenta and cord blood (p<0.05).

3 Placenta with higher percentage of NKTs than cord blood (p = 0.005). HCV Placenta higher than Control placenta (p = 0.05). Control placenta less than control decidua (p = 0.005). PBMC NKTs higher than HCV and control cord blood (p≤0.03).

4 Decidua B Lymphocytes higher than cord blood (p<0.05). HCV cord blood higher than control cord blood. (p<0.05).

NA  =  Not assessed; PBMCs were available for HCV-infected mothers only. Median and range are shown.

### Dendritic cells

**Figure 1:**
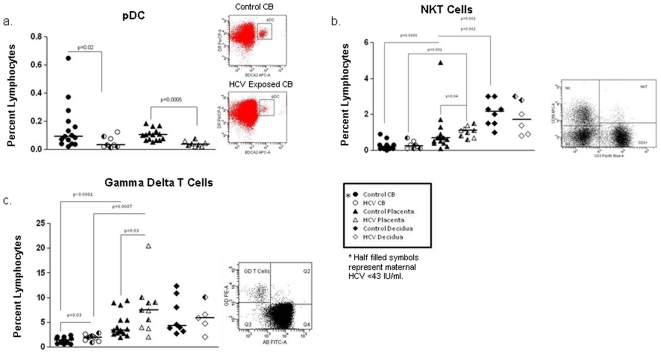
Innate lymphocyte composition of cord blood, placenta and decidua in control and HCV dyads. Plasmacytoid dendritic cells (pDC) were detected at similar levels in cord blood and placenta. Decreased pDCs were observed in HCV-exposed subjects compared to healthy controls. Representative flow dot plots for cord blood are shown (a). NKT cells were elevated in decidua compared to placenta and in placenta compared to cord blood. NKT cells are increased in HCV-exposed placenta compared to controls. Multiparameter flow analysis dot plot demonstrating identification of NK, and NKT cell populations (b). Gamma Delta (γδ) T cells are increased in placenta compared to cord blood. HCV-exposed cord blood and placentas had increased γδ T cells compared to healthy controls. Representative flow analysis plot demonstrating γδ T cells in HCV-exposed cord blood (c).

Dendritic cells (DC) (Lin^−^DR^+^BDCA^+^) link the innate and adaptive immune responses and are known to be decreased in number and function during HCV infection [24]–[27]. During pregnancy, DCs have been shown to display decreased activity in decidua tissue as a mechanism of increased tolerance of fetal antigens; however, DCs have not previously been evaluated in placental tissue [28], [29]. We found plasmacytoid DCs (pDC) to be decreased in HCV-exposed infants compared to controls, both in CB (p = 0.02) and placenta (p = 0.0005) (**Figure 1a**). No differences were seen between CB and placenta in both controls and HCV-exposed or between HCV-exposed and controls subjects in the myeloid dendritic cell (mDC) population. Due to limited numbers of available cells, DCs were not evaluated in maternal decidua or PBMCs.

### NKT and gamma delta T cells

Conventional T cells expressing clonotypic receptors (TCRs) which recognize peptide fragments of protein antigens complexed with MHC class I (CD8^+^ cytotoxic T cells) or class II (CD4^+^ helper T cells) molecules are central players in adaptive immune responses [13], [30]. However, diverse populations of T cells express antigen receptors of limited diversity that are not restricted by conventional polymorphic MHC molecules [13]. These non-conventional T cells have antigen recognition mechanisms, cytokine profiles and responses to stimulation which differ significantly from those of conventional T-cell populations and some of which are shared with NK cells. Thus, they are thought to represent important innate immune effector populations likely to be important in the first line of defense against viral and other pathogens [31]. The majority of non-conventional T cells also express NK activating and inhibitory receptors and are termed NKT cells. A small subset of NKT expresses an invariant TCR consisting of the Vα24JαQ-chain with a limited number of β-chains (Vβ8 or Vβ11) [32], [33]. The majority of conventional T cells express the αβ-TCR, however, approximately 5% of peripheral T cells express an alternative γδ*-*TCR [34]. They possess oligoclonal or invariant TCRs that recognize a wide array of antigens including soluble non-peptide antigens and stress-inducible proteins without the need for MHC restriction and accumulate at sites of infection [34].

**Figure 2:**
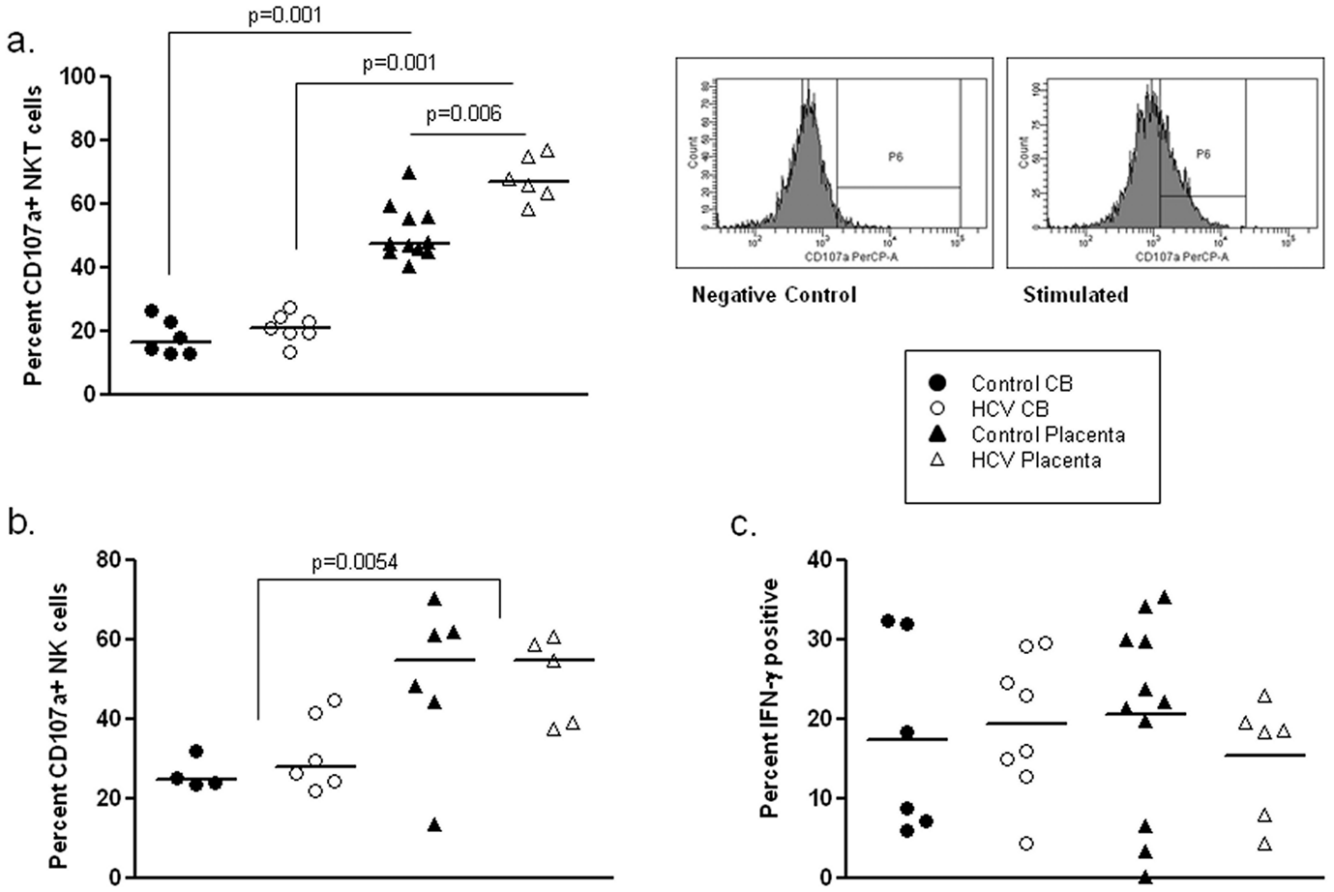
NKT and NK cell cytotoxicity. NKT and NK cell cytotoxicity was measured using flow cytometric analysis of CD107a after stimulation as described in the methods section. NKT cell cytotoxicity was increased in placenta compared to cord blood and is further amplified following HCV exposure. Representative CD107a histogram on placenta (a). NK cell cytotoxicity is increased in placenta compared to cord blood (b). NK cell production of IFN-γ is robust in all compartments (c).

Early HCV infection is associated with a decrease in NKT cells [35], [36]. Spontaneous recovery from HCV infection is associated with NKT cells that demonstrate a more activated phenotype [36]. NKT cells are known to be increased in decidua with a proposed role in the Th1/Th2 balance [20]. In our study, NKT cells are higher in control decidua than in placenta (p = 0.02). Both control and HCV-exposed placenta demonstrate higher proportional frequency of NKT than CB (p = 0.0007 and p = 0.008). NKT cells were increased in HCV exposed placenta compared to control placenta (p = 0.04) (**Figure 1b**). Moreover, the cytotoxicity of NKT cells (assessed by CD107a expression) was greater in placenta than CB and was further increased in HCV-exposed dyads (**Figure 2a**). Invariant NKT cells (Vα24^+^Vβ11^+^) are present in both CB and placenta, but are less than 1% of lymphocytes in both controls and HCV exposed.

Gamma Delta (γδ) T cells comprise another innate immune population likely to play a significant role in protection from viral infection [37]. With a proposed role in protection from intrauterine infection [38] during pregnancy, γδ-T cells are known to be increased in healthy term decidua compared to first trimester decidua. As expected, we found γδ-T cells are more prevalent in placenta than CB (p = 0.0005). Furthermore, HCV exposure increased the presence of γδ*-*T cells in both CB and placenta (p = 0.03, p = 0.03) (**Figure 1c**).

### NK cell frequency, phenotype and function

Impairment of NK cells (decreased number and function) in acute HCV has been shown to lead to chronic HCV [3], [39]. In HCV infection, the impaired NK cells may be the result of decreased IFN-α production by pDC resulting in impaired NK cell and T-cell activation [40]–[42].

NK (CD3^−^CD56^+^) cells are central to the innate immune system [42]–[46]. In HCV infection, NK cells in the peripheral blood are decreased and subset frequencies are altered, e.g., decreased CD56^dim^ or the so-called effector NK cells, and decreased cytokine production [39], [44]. In addition, HCV infection has been shown to alter NK cell surface receptors and NK cell functionality leading to chronic infection [30]. During pregnancy, NK cells are known to predominate maternal decidua with a CD56^bright^ phenotype; however no analysis has been completed on placental tissue [19], [47]. We found that CD56^bright^ NK cells were more frequent in the decidua as compared to placenta (Supplemental **Figure S1**). NK cells, as a percentage of total lymphocytes, trended higher in decidua compared to placenta with no effect from HCV exposure (**Figure S1**).

As NK cells represent one of the primary anti-viral innate immune effector population likely to be involved in protection from HCV acquisition in the face of exposure, we tested the hypothesis that heightened NK cell activity would be protective, perhaps accounting for the low rate of vertical transmission from an infected mother. We directly ex vivo characterized the function and phenotype of NK cell populations from CB and placenta as well as term decidua. Cytotoxicity was evaluated as an indication of NK cell function using the CD107a degranulation assay. Following stimulation with PMA and Ionomycin, placenta demonstrated greater cytotoxicity than CB (p = 0.0054) (**Figure 2b**). As an additional functional correlate, cytokine production was assessed. CB is known to have decreased cytokine production compared to peripheral blood [23]. No differences in IFN-γ (**Figure 2c**) or TNF-α (data not shown) production were seen between placenta and CB in controls versus the HCV-exposed. It is interesting to note that robust IFN-γ production was seen in both placenta and cord blood despite HCV exposure.

**Figure 3:**
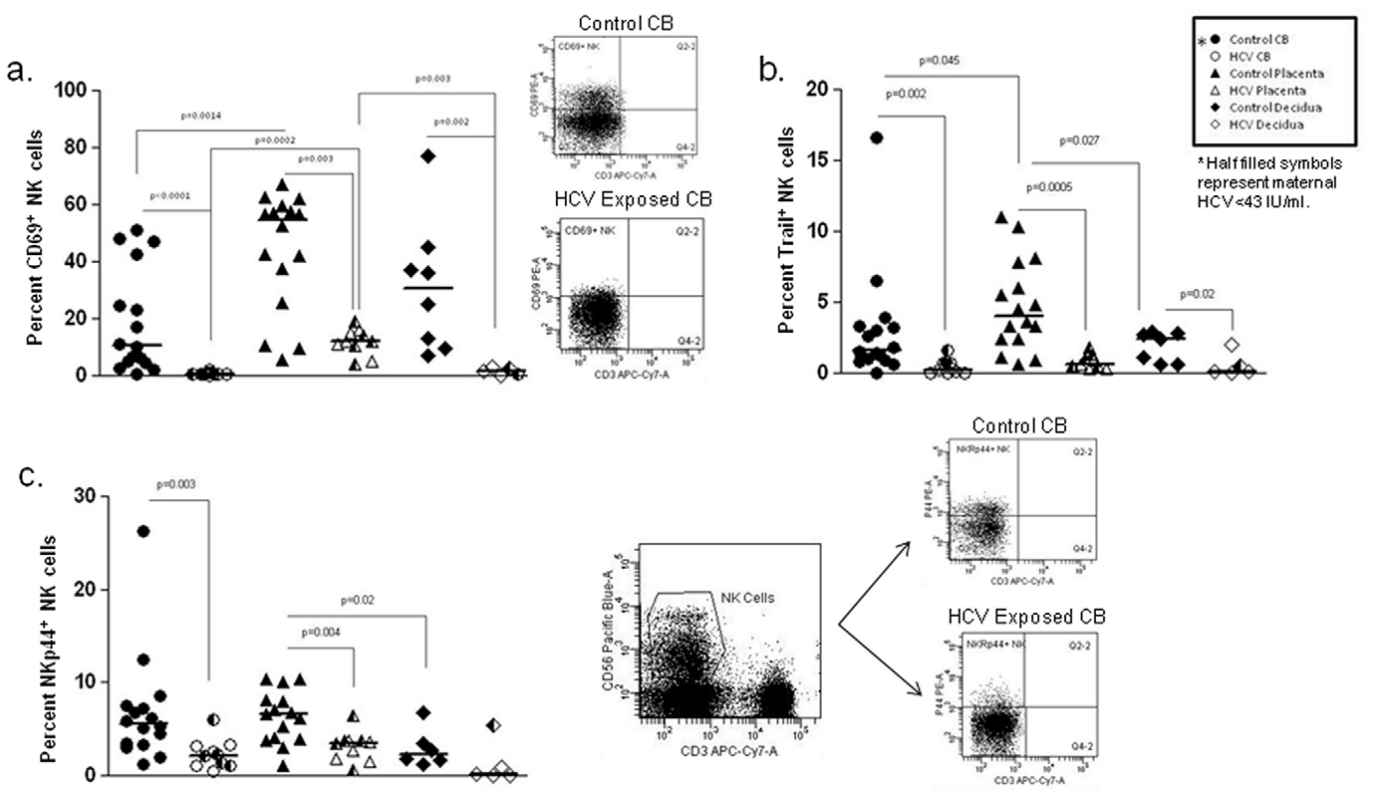
NK cell surface receptor expression. Placenta and decidua NK cells express elevated CD69 compared to cord blood. HCV exposure results in decreased NK cell CD69 expression in all tissues. Representative plots of CD69 expression on cord blood are shown (a). Placenta NK cells have increased TRAIL compared to cord blood and decidua. TRAIL is decreased in HCV exposure in all tissues (b). NKp44 expression trends higher in placenta NK cells compared to decidua. NKp44 expressing NK cells are decreased on HCV exposure. Representative multiparameter flow analysis of HCV-exposed and control cord blood NKp44 expression on NK cells is shown (c).

NK cells use a variety of cell surface receptors to control their activation, effector functions and even proliferation [19]. Following the identification of increased cytotoxicity in placenta, we evaluated the phenotype of the NK cells to identify any markers of increased activation or decreased inhibition in this population. The phenotype of NK cells was evaluated with particular attention to activating receptors CD69, TRAIL and NKp44 (**Figure 3** **and** supplemental **Table S1**). In addition NKG2A, NKG2C, NKG2D, CD158a, CD158b, NKp46, NKp30 and CD161 were also assessed (supplemental **Table S2**).

CD69 is an early activation marker expressed on a number of cell types. In HCV infection, CD69 on peripheral NK cells (CD56^dim^ subset) has been shown to correlate with serum ALT as surrogate marker of liver necroinflammation and its expression on the CD56^bright^ subset correlated with serum HCV RNA levels [48]. In pregnancy, CD69^+^ NK cells have previously been shown to be elevated in decidua compared to maternal peripheral blood [46]. In our current study, we found higher proportions of CD69^+^ NK cells in both control and HCV-exposed placenta than in CB (p = 0.02 and p = 0.003) and compared to HCV decidua (p = 0.04) (**Figure 3a**). In contrast to the finding that the frequency of CD69^+^ NK cells in the peripheral blood does not differ between normal controls and HCV-infected patients [48], we found decreased numbers of CD69^+^ NK cells in CB, placenta, and maternal decidua exposed to HCV (**Figure 3a**). This pattern was consistent when examining the CD56^bright^ and CD56^dim^ populations (data not shown).

Tumor necrosis factor-related apoptosis-inducing ligand (TRAIL), is a cytotoxicity receptor and another activation marker found on NK cells [49], [50]. TRAIL has been described to be increased in chronic HCV [51]. TRAIL has not been evaluated on placenta. In our dyads, TRAIL was lower in NK cells of CB and placenta in the HCV exposed infant group (p = 0.002 and p = 0.0005) and lower in decidua of HCV infected women (p = 0.02). Additionally, TRAIL was higher in placenta than in CB and decidua of controls (p = 0.05 and p = 0.05) (**Figure 3b**).

Dysregulated expression of natural cytotoxicity receptors (NCR; e.g., NKp30, NKp44, and NKp46) in HCV infection have been described by a number of groups [52]. NKp44 mediates activation of NK cells and is increased in peripheral blood of adults who clear HCV infection [52]. During pregnancy, decidua NK cells express NKp44 with a strong correlation with cytokine production and cytotoxicity [53]. In contrast, maternal peripheral blood NK cells are devoid of NKp44 [53]. In our study, NKp44 is identified at lower frequency in HCV-exposed CB and placenta (p = 0.003 and p = 0.004). In addition, NKp44 trends lower in control decidua than in control placenta (**Figure 3c**). No consistent differences were seen in the relative expression of NKG2A, NKG2C, NKG2D, CD158a, CD158b, NKp46, NKp30 and CD161 (**Table S2**). Maternal PBMCs were also compared with differences only in CD158b (**Table S2**).

## Discussion

Early inhibition of innate immunity contributes to the development of persistence in HCV infection [54]. In order to define the role placental immune function plays in protection from vertical transmission of HCV infection, we performed the first thorough characterization of innate cells on term placenta paired with CB and decidua using multiparameter flow cytometry. The principal findings of this study can be summarized as follows: 1) the overall lymphocyte composition of placenta is remarkably similar to CB and decidua, although NK and NKT cells are found at greater frequencies within decidua than placenta or CB; 2) plasmacytoid DCs are decreased in the CB and placenta of HCV-exposed infants; 3) NK cells demonstrated greater activation (CD69-positivity) in the placenta than in decidua or cord blood; 4) HCV exposure increases the proportion of NKT cells and γδ-T cells in the placenta; 5) HCV infection leads to decreased numbers of CD69^+^ NK cells within the three compartments (maternal decidua, placenta, and CB), as well as lower expression of TRAIL and NKp44, also involved in activation; 6) robust IFN-γ production by NK cells is detectable in all three compartments and NK cytotoxicity is greater in placenta than CB. Our analyses of placental lymphocyte composition and phenotype support the concept that the placenta and decidua protect the infant from viral infiltration.

The differences in innate immune composition include increased NKT cells in placenta compared to cord blood, in keeping with previous studies documenting increased NKT cells in maternal decidua compared to maternal peripheral blood [20]. It has been proposed that the NKT cells play a significant role in determining the Th1/Th2 balance in decidua and it is likely they have a similar function in placenta [20]. The ability of NKT cells to quickly produce cytokines and therefore stimulate the innate immune response could explain their increased presence in placenta and decidua tissue. In addition to increased NKT cells, γδ*-*T cells are increased in placenta compared to CB. This again parallels the increases seen in decidua [38]. Further, our analysis supported the previous findings of elevated NK (CD3^−^CD56^+^) cells in the decidua [19], [47]. It is interesting to note the increase is not also seen in placenta tissue. While the control placenta NK cells are not increased compared to CB, they do demonstrate a more activated phenotype with elevated NKG2C, CD69 and TRAIL (**Figure 3** **and Table S2**). More importantly, the cytotoxicity of placenta is higher than CB. Taken together, these data support a conceptual paradigm with a relative gradient of NK, NKT, and γδ-T cells to maximize antiviral protection for the infant.

HCV has been shown to dysregulate a number of components of the host immune response, but its effect on the placental-fetal microenvironment had not been previously characterized. Circulating pDC have been shown to demonstrate diminished IFN-α production, potentially impairing T-cell and NK-cell activation [40], [41]. In keeping with these studies in adult peripheral blood, we found that pDC were decreased in number in both CB and placenta in HCV-exposed infants.

NKT and γδ-T cells are non-conventional T cells that likely provide an important first line of defense against infectious pathogens. NKT cells have been shown to be important for clearance of HCV in the acute setting [30] and a decrease in intrahepatic NKTs has been associated with more severe pathology in chronic HCV infection [35]. γδ-T cells are part of the innate immune response and are widely distributed in peripheral blood and epithelial surfaces of the skin, lung, and reproductive tract and gut. γδ-T cells are known to be important in the initial viral control of Herpes Simplex Virus, Varicella, and West Nile virus (a flavivirus like HCV) in mouse models [35], [54], [55]. The fact there is enrichment of NKT and γδ-T cells in placenta (relative to CB), and this was increased further in HCV-exposed dyads might provide an explanation for the low rate of HCV vertical transmission.

HCV infection has been shown to alter NK cell surface receptors and NK cell functionality leading to chronic infection [30]. We found that HCV infection decreased the relative expression of activation markers CD69, TRAIL and NKp44 in decidua, placenta and CB. Despite these phenotypic changes, cytokine production by NK cells remained robust and NKT cytotoxicity was increased in HCV infection. These results represent a potential mechanism by which the placenta prevents vertical transmission during pregnancy, and because infection elicits NKT cytotoxicity, a mechanism that may contribute to the observed increase in preterm labor in HCV infection [10]. Of the 16 mothers identified for the study, 3 (18%) had premature delivery resulting in two fetal losses and one 24 week infant.

In summary, this study identifies novel data related to the differential frequency and phenotype of innate immune cells in paired placental-cord blood samples and provides the first formal demonstration that HCV affects the immunobiology of the maternal-fetal interface. Work is ongoing to define how innate immune responses shape adaptive immune responses and protect against vertical transmission.

## Materials and Methods

### Ethics Statement

The study protocol was approved by the Institutional Review Boards at the University of Colorado and Denver Health Medical Centre. Both written and oral consent was obtained before samples were collected.

### Study Population

The study group was comprised of HCV exposed (n = 12) and healthy control (n = 16) mother infant pairs recruited from two sites. All HCV infected mothers were HCV antibody positive prior to enrollment with no treatment for HCV prior to or during pregnancy. The study protocol was approved by the appropriate institutional review board. Informed consent was obtained from each subject.

### Sample preparation and storage

Placental tissue (average 100 grams) was collected immediately following delivery. The maternal basal plate (decidua) was manually dissected from the fetal placental tissue. After separation the tissue was mechanically disrupted and enzymatically digested with collagenase 1A (Sigma Aldrich, St Louis, MO) for 60 minutes on a rocker at 37°C. Placental and decidual mononuclear cells were collected by Ficoll (Amersham Biosciences, Piscataway, NJ) density gradient centrifugation. The mean placental sample yielded 180 million mononuclear cells. Isolated mononuclear cells were then cryopreserved (20% dimethyl sulfoxide in fetal bovine serum) for subsequent analysis. Cord blood (80–125 ml) was collected at the time of delivery. Mononuclear cells from cord blood were collected by density gradient centrifugation and stored as above with an average yield of 120 million mononuclear cells. Cell viability was confirmed by trypan blue staining. Dead cells were excluded following thawing utilizing appropriate forward and side scatter selection on flow cytometry, per the laboratory standard protocol.

### Flow cytometric analysis

Multiparameter flow cytometry was performed using a BD FACSCanto II instrument (BD Biosciences, San Jose, CA) compensated with single fluorochromes and analyzed using FACSDiva software (BD Biosciences).

### Cell surface Staining and antibodies

Fluorochrome-labeled monoclonal antibodies (MAb) specific for TCR αβ/γδ, CD3, CD4, CD8, CD14, CD19, CD56, CD158a, CD161, HLA-DR, CD69, CD45RA, Lin, NKp46 and NKp30, were purchased from BD Biosciences (San Jose, CA). Anti-TRAIL, and anti-NKG2A/C/D antibodies were supplied by R&D Systems (Minneapolis, MN) and anti-Vβ11 by Beckman Coulter (Brea, CA). Anti-Vα24 was obtained from Immunotech (Prague, Czech Republic), CD158b and anti-BDCA-2 (pDCs), BDCA-1 (mDCs) MAbs from Miltenyi (Auburn, CA). Thawed fetal cord blood, maternal PBMC from HCV-infected mothers, fetal placental and maternal decidual mononuclear cells were stained for cell surface antigen expression at 4°C in the dark for 30 min, washed twice in 2 ml phosphate-buffered saline containing 1% bovine serum albumin and 0.01% sodium azide (fluorescence-activated cell sorter wash), and subsequently fixed in 200 µl of stabilizing fixative (BD). Isotype-matched control antibodies were used to determine background levels of staining.

### Cytokines/Intracellular Staining

Cytokine production by cord blood mononuclear cells and placental mononuclear cells was analyzed after a 6 hour stimulation with PMA (10 ng/ml, Sigma-Aldrich) and Ionomycin (1 ug/ml, Sigma-Aldrich) at 37°C. Golgi Stop solution (BD Bioscience, San Jose, CA) was added to each sample. Intracellular cytokine staining (ICS) was carried out using Caltag solutions A and B (Fix and Perm solutions) according to the manufacturer's instructions (Caltag, Burlingame, CA). Anti- TNF-α and IFN-γ antibodies were purchased from BD Biosciences.

### Degranulation Assay

CD107a (LAMP) degranulation assay was performed as a well accepted indirect measure of cytotoxicity. Cord blood and placental mononuclear cells were stimulated, as above (PMA), for 1 hour and then incubated for 5 hours with GolgiStop in the presence of anti-CD107a-PerCP antibody from BD Biosciences (San Jose, CA). Degranulation was determined by flow cytometric analysis of increased CD107a expression on gated NK cell populations (CD3^−^CD56^+^). Cells cultured under the same conditions in absence of stimulation served as controls.

### Statistical analyses

Results are expressed as median (range). Mann-Whitney U test or Wilcoxon Matched Pairs test were used to compare differences between patient groups as appropriate with a significance level of 0.05. Comparisons were made between the physiologic pairs of cord blood and placenta and, decidua and placenta to minimize comparisons. No adjustment for multiple comparisons was used as this was an exploratory study aimed at hypothesis generation. Decidua and cord blood were only compared for NK cell percentages given the significant existing data on decidua. Prism 5 by Graphpad (San Diego, CA) statistical and graphing package was used.

## Supporting Information

Figure S1 NK cell and CD56^bright^ NK cells in placenta and decidua. NK cells trend higher in decidua compared to placenta (a). CD56^bright^ NK cells trend higher in control decidua compared to placenta (b).
(0.67 MB TIF)

Table S1 Innate immune cell lines and cell surface markers of interest (median and range).
(0.04 MB DOC)

Table S2 Stimulatory and inhibitory NK cell surface markers Percent of lymphocytes (median and range). *NKG2C is higher in control placenta compared to cord blood. (p = 0.01) ∧NKG2D is lower in control placenta compared to cord blood (p = 0.001) and decidua (p = 0.002). HCV exposure results in increased NKG2D (p = 0.01) +CD158b in increased in control placenta compared to cord blood (p = 0.0004) and in HCV placenta compared to HCV cord blood (p = 0.02). CD158b is higher in HCV PBMC compared to both HCV cord blood and control cord blood (p<0.03). &Control placenta has lower NKp46 compared to control cord blood (p = 0.007) ¥ Control placenta is lower than control cord blood (p = 0.0002) and control decidua (p = 0.005) ∏ Control placenta is lower in CD161 compared to control cord blood (p = 0.0006) and control decidua (p = 0.02).
(0.04 MB DOC)
