# Supplementary figures and images for: Innate Immune Function in Placenta and Cord Blood of Hepatitis C – Seropositive Mother-Infant Dyads
Source: PLoS One. 2010 Aug 30;5(8):e12232. doi: 10.1371/journal.pone.0012232 (PMC2923602; doi:10.1371/journal.pone.0012232)

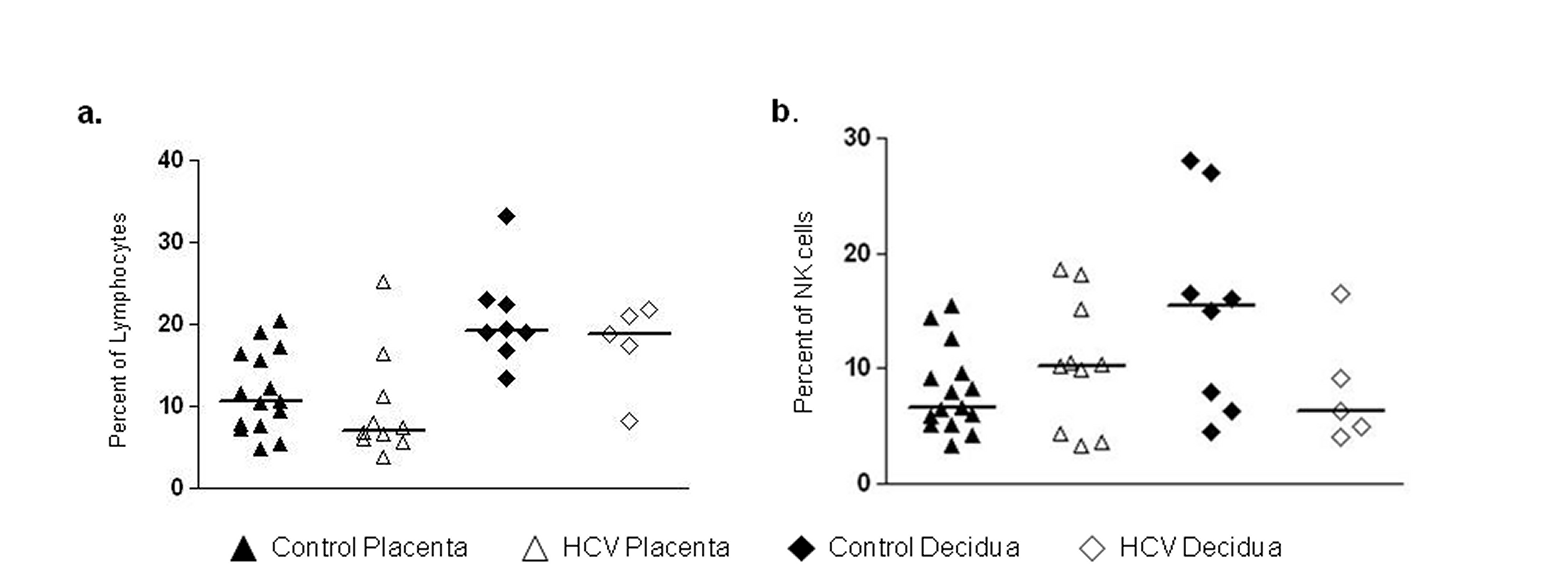

Supplement: Figure S1 — NK cell and CD56bright NK cells in placenta and decidua. NK cells trend higher in decidua compared to placenta (a). CD56bright NK cells trend higher in control decidua compared to placenta (b). (0.67 MB TIF) [file pone.0012232.s001.tif]
