# Supplementary material for: Innate Immune Function in Placenta and Cord Blood of Hepatitis C – Seropositive Mother-Infant Dyads
Source: PLoS One. 2010 Aug 30;5(8):e12232. doi: 10.1371/journal.pone.0012232 (PMC2923602; doi:10.1371/journal.pone.0012232)
Supplement: Table S1 — Innate immune cell lines and cell surface markers of interest (median and range). (0.04 MB DOC) [file pone.0012232.s002.doc]

|  | **pDC** | **γδ-T cells** | **CD69^+^ NK cells** | **TRAIL^+^ NK cells** | **NKp44^+^ NK cells** |
| --- | --- | --- | --- | --- | --- |
| Control Cord Blood | 0.091% (0.021-0.37) | 1.35% (0.6-2.4) | 10.6% (0.8-51.2) | 1.65% (0-16.6) | 5.6% (1.2-26.3) |
| HCV Cord Blood | 0.104 (0.053-0.186) | 3.4 (1.9-8.5) | 54.4 (5.6-67.3) | 4.05 (0.6-10.3) | 6.6 (3.7-10.4) |
| HCV PBMC | NA* | 5.9 (1.9-7.6) | 1.2 (0.2-6.7) | 0.5 (0-2) | 0.1 (0-0.2) |
| Control Placenta | 0.031 (0.014-0.123) | 1.95 (0.9-2.8) | 0.75 (0.4-2) | 0.25 (0-1.6) | 2.2 (0.5-6.1) |
| HCV Placenta | 0.036 (0.02-0.077) | 7.45 (2.0-20.5) | 12.0 (4.3-19.1) | 0.6 (0.3-1.8) | 3.5 (0.6-6.5) |
| Control Decidua | NA | 4.3 (2.7-12.4) | 30.8 (9.4-77.2) | 2.4 (0.6-2.9) | 2.3 (1.2-6.8) |
| HCV Decidua | NA | 59.0 (2.1-10.1) | 1.4 (0.3-3.2) | 0.1 (0-0.5) | 0.6 (0-5.4) |

*NA = Not assessed

**Table S1.** Innate immune cell lines and cell surface markers of interest (median and range).
