# Supplementary material for: Innate Immune Function in Placenta and Cord Blood of Hepatitis C – Seropositive Mother-Infant Dyads
Source: PLoS One. 2010 Aug 30;5(8):e12232. doi: 10.1371/journal.pone.0012232 (PMC2923602; doi:10.1371/journal.pone.0012232)
Supplement: Table S2 — Stimulatory and inhibitory NK cell surface markers Percent of lymphocytes (median and range). *NKG2C is higher in control placenta compared to cord blood. (p = 0.01) ∧NKG2D is lower in control placenta compared to cord blood (p = 0.001) and decidua (p = 0.002). HCV exposure results in increased NKG2D (p = 0.01) +CD158b in increased in control placenta compared to cord blood (p = 0.0004) and in HCV placenta compared to HCV cord blood (p = 0.02). CD158b is higher in HCV PBMC compared to both HCV cord blood and control cord blood (p<0.03). &Control placenta has lower NKp46 compared to control cord blood (p = 0.007) ¥ Control placenta is lower than control cord blood (p = 0.0002) and control decidua (p = 0.005) ∏ Control placenta is lower in CD161 compared to control cord blood (p = 0.0006) and control decidua (p = 0.02). (0.04 MB DOC) [file pone.0012232.s003.doc]

|  | **NKG2A** | **NKG2C** | **NKG2D** | **CD158a** | **CD158b** | **NKp46** | **NKp30** | **CD161** |
| --- | --- | --- | --- | --- | --- | --- | --- | --- |
| Control Cord Blood | 15.4% (1.5-61.9) | 4.4% (1.8-10.8) | 91.5% (83.8-98.0) | 19.8% (13.0-48.3) | 11.2% (7.1-71.4) | 80.0% (30.6-96.6) | 85.9% (79.0-97.7) | 88.2% (21.1-95.2) |
| HCV Cord Blood | 10.7 (5.0-31.5) | 3.7 (1.6-56.4) | 87.1 (5.3-95.8) | 20.8 (7.6-57.6) | 20.7 (12.6-83.1) | 74.1 (24.2-91.4) | 92.4 (0.1-94.4) | 73.0 (2.4-95.1) |
| HCV PBMC | NA | NA | 41.5 (30.4-55) | 28.7 (16.7-49.9) | 34.1 (23.4-51.1) | 64.9 (23.4-91.7) | 79.5 (39.3-94.7) | 78.9 (67.3-90.1) |
| Control Placenta | 23.0 (11.5-47.5) | 13.1***** (2.8-40.9) | 8.9^ (0.6-92.4) | 22.8 (8.6-50.2) | 37.9+ (5.6-25.8) | 52.2& (24.2-91.1) | 64.8**^¥^** (6.5-91.6) | 34.3**^π^** (2.7-76.9) |
| HCV Placenta | 21.2 (9.2-49.9) | 7.5 (0.2-20.7) | 88.6 (53.0-98.3) | 23.6 (12.0-50.8) | 26.8 (13.2-47.8) | 39.8 (9.8-94.9) | 73.4 (33.3-86.7) | 56.8 (29.1-87.6) |
| Control Decidua | NA | NA | 98.4 (86.5-99.0) | 18.0 (5.6-40.3) | 30.5 (17.1-49.5) | 58.0 (20.9-93.4) | 91.4 (74.0-97.9) | 87.8 (0.4-97.3) |
| HCV Decidua | NA | NA | 93.2 (70.8-98.4) | 22.5 (9.6-31.6) | 34.8 (28.1-74.2) | 88.7 (4.3-97.6) | 95.0 (7.8-96.7) | 91.5 (2.1-95.1) |

NA = Not assessed

**Table S2**. Stimulatory and inhibitory NK cell surface markers

Percent of lymphocytes (median and range).

*****NKG2C is higher in control placenta compared to cord blood. (p=0.01)

**^**NKG2D is lower in control placenta compared to cord blood (p=0.001) and decidua (p=0.002). HCV exposure results in increased NKG2D (p=0.01)

^+^CD158b in increased in control placenta compared to cord blood (p=0.0004) and in HCV placenta compared to HCV cord blood (p=0.02). CD158b is higher in HCV PBMC compared to both HCV cord blood and control cord blood (p<0.03).

**^&^**Control placenta has lower NKp46 compared to control cord blood (p=0.007)

**^¥^** Control placenta is lower than control cord blood (p=0.0002) and control decidua (p=0.005)

^Π^ Control placenta is lower in CD161 compared to control cord blood (p=0.0006) and control decidua (p=0.02).
